# Management of simultaneous ocular elevation and depression deficit in patients after reconstruction surgery for orbital floor fracture

**DOI:** 10.1007/s00417-020-04659-y

**Published:** 2020-04-15

**Authors:** Piotr Loba, Agata Joanna Ordon

**Affiliations:** Department of Binocular Vision Pathophysiology and Strabismus, Medical University of Lodz, University Barlicki Hospital No.1, Kopcinskiego Street 22, 90-153 Lodz, Poland

**Keywords:** Surgical reconstruction, Orbital fracture, Diplopia, Ocular motility deficit

## Abstract

**Purpose:**

To present and examine the results of surgical correction of simultaneous ocular elevation and depression deficit in patients who underwent reconstruction surgery for orbital floor fracture.

**Methods:**

A retrospective analysis of medical records of patients who had undergone surgical correction for diplopia associated with orbital fracture which persisted after orbital reconstruction surgery. All patients underwent orthoptic evaluation before surgery and postoperatively with various times of follow-up.

**Results:**

Eight cases of blow-out fracture of the orbital floor were identified. Surgical plan varied from case to case. It included thorough revision of inferior rectus/oblique complex with or without recession of the former or flap tear repair and additional procedures. Postoperatively 4 patients (50%) were diplopia free, 3 (37.5%) presented diplopia in extreme upgaze and 1 (12.5%) in mid-upgaze and adduction. None of the patients reported diplopia in the primary position neither downgaze.

**Conclusion:**

Diplopia persisting after reconstructive surgery of a fractured orbital floor may be corrected surgically. Our results suggest that at least two surgical procedures are necessary to achieve satisfying outcomes. Contralateral inferior rectus recession combined with superior oblique recession and superior rectus posterior fixation appears to be effective procedures for use.

## Introduction

Despite precise reconstruction of orbital floor fracture performed in order to restore orbital anatomy and volume, patients may be affected by some serious long-term sequelae [1] that require surgical treatment [2].

Potential complications following the orbital floor fracture repair may include incomplete correction of preoperative enophthalmos and diplopia, as well as induction of the globe dystopia, eyelid malposition, or optic nerve injury [3]. Residual diplopia is regarded as the most common post-treatment complication of orbital bone fracture reduction [4, 5].

Disturbances of ocular motility found in such patients may be caused by direct trauma to the orbital content, including the extraocular muscles and surrounding adnexal tissues, leading to diplopia and loss of binocular vision [6].

In general, ocular motility impairment may have two possible mechanisms: functional disability due to paralysis of the extraocular muscles or their mechanical restriction by nearby structures [7]. Moreover, posttraumatic ocular motility disturbances may present as several different patterns. The most common is limited elevation of the globe. Surgical management of diplopia in upgaze was described in one of our previous papers [8]. However, in some cases, it is accompanied by more or less severe eye depression deficit. In such instance, the patient reports diplopia in both up- and downgaze. This is usually due to the weakness of the inferior rectus muscle caused by denervation, direct trauma, or posterior synechiae [9]. Such pattern of ocular motility limitation poses a huge challenge for a managing strabologist. Proper surgical approach is crucial for both functional and cosmetic aspects. The motility patterns, as well as the complaint of the patients and their expectations must be taken into consideration.

The major objective of this study was to present and retrospectively analyze the results of surgical correction of eye’s simultaneous elevation and depression deficit in patients who previously underwent reconstruction surgery for orbital floor fracture.

## Methods

A retrospective case review of patients referred to the Department of Binocular Vision Pathophysiology and Strabismus, Medical University of Lodz, was performed over a period of 7 years. The study group consisted of patients that had undergone surgical correction for diplopia associated with orbital fracture, persisting after orbital reconstruction surgery. The study was restricted only to those cases in which diplopia was significant in both upgaze and downgaze or downgaze diplopia appeared as a consequence of previous weakening procedure on a restricted inferior rectus muscle, ipsilateral to the fractured orbit. Data concerning type of fracture, timing of reconstruction surgery, alloplastic materials used, number and timing of the strabismus surgeries were evaluated. All patients underwent orthoptic evaluation before surgery and postoperatively with various times of follow-up. Ocular rotations, the type and direction of diplopia, and the vertical angle of strabismus measured by prism and cover test were noted in prism diopters [Δ]. Hess charts were plotted in each case as well as Binocular Single Vision Field (BSV) (Medmont M900).

The results were subject to statistical analysis (STATISTICA 13.1) using *t* test for paired measurements (*p* < 0.01). The methods applied in the study adhered to the Declaration of Helsinki and were accepted by the Board of Ethics of the Medical University of Lodz (RNN/144/09/KE).

## Results

Eight cases (5 male, 3 female) were identified. The mean age of patients was 34.1 ± 7.2 years. The time difference between the orbital trauma and reconstruction surgery ranged from 7 to 90 days with a mean of 34.6 ± 31 days. The orbital floor fracture was present in all of the cases with 5 (62.5%), orbital rim was additionally involved in 3 patients (37.5%). The characteristics of the reconstruction surgery performed in this group of patients are summarized in Table 1.

**Table 1:** Study group characteristic (*n* = 8)

Side of the fracture	
Right	3 (37.5%)
Left	5 (62.5%)
Type of implant	
Polipropylen sheet	3 (37.5%)
Titanium mesh	5 (62.5%)
Time from trauma to reconstruction surgery	
≤ 2 weeks	5 (62.5%)
> 2 weeks	3 (37.5%)
Additional procedures after reconstruction	
Implant repositioning	2 (25%)
Secondary inlay implant	1 (12.5%)

Mean time that elapsed from the reconstruction surgery to the first strabismus procedure was 10.3 ± 5.5 months (6 to 24 months). During each strabismus surgery, a forced duction test was performed under general anesthesia in order to confirm a restriction of inferior rectus muscle. Final check-up was conducted after a mean follow-up of 13 months, range from 7 to 24 months.

In our study, the surgical correction of diplopia consisted of at least two procedures. Except one patient (case 1), the first move was to revise the inferior rectus muscle and surrounding area for possible synechiae, flap tears or avulsion. In two cases (7 and 8), a flap tear was identified freed from the surrounding adhesions and resewn. In other two cases (5 and 6), the inferior rectus muscle was recessed after freeing it from adhesions. This was done due to a still limited passive elevation. Subsequently, in all but one above cases, an area of double vision was reduced in upgaze but appeared in downgaze as a result of inferior rectus weakening. In other three cases (2, 3, and 4), the firm synechie between the periorbita and inferior rectus and oblique muscles were dissected, but no recession was done. Those patients reported diplopia in both vertical directions preoperatively. The first procedure resulted in a slight improvement of ocular motility in upgaze but did not alleviate diplopia in downgaze.

In only one case (case 8), the first stage surgery resulted in improvement of ocular motility in downgaze (as a result of a flap tear repair) to an extent that the remaining problem was limited elevation. That’s why the second step in this case was superior rectus posterior fixation suture. In other six cases, the second step was warranted.

The second step in cases 2–7 was similar and consisted of simultaneous contralateral superior rectus faden procedure with or without recession, inferior rectus, and superior oblique recessions.

In one case, additional step was performed. In case 3, the final result was prominent undercorrection in upgaze resulting from contralateral inferior oblique overaction. We have performed inferior oblique recession 12 mm in order to correct it.

Case 1 was the first in our series and we did not start from revising the inferior rectus/oblique complex but straight went to the contralateral eye for superior oblique tenectomy and inferior rectus recession in order to correct diplopia in downgaze which was most disturbing for the patient. Subsequently, we achieved a great improvement in the mentioned gaze but diplopia remained in upgaze. The faden procedure on superior rectus was performed as a second step procedure. Table 2 shows an overview of strabismus procedures performed at each step of surgical treatment.

**Table 2:** Angles of vertical deviation at presentation and after each surgical procedure

Patient	Angle at presentation (∆)	1 Step	Angle after the first step (∆)	2 Step	Final angle (∆)
1	↑ RHT^a^ 14	RE: rec IR 5.5 mm; SO tenectomy	↑ RHT 24	RE: SR faden + rec SR 3,0 mm	↑ RHT 4
	• LHT 8		• LHT 16		• 0
	↓ LHT 20		↓ LHT 4		↓ LHT 4
2	↑ RHT 10	LE: rev IR/IO	↑ RHT 8	RE: rec IR 5.0 mm; rec SO; SR faden	↑ RHT 6
	• LHT 6		• LHT 8		• 0
	↓ LHT 22		↓ LHT 22		↓ LHT 2
3	↑ RHT 12	LE: rev IR/IO	↑LHT 12	RE: rec IR 5.5 mm; rec SO; SR faden//3.step**//** RE: IO rec 12.0 mm	↑ RHT 12
	• LHT 16		• LHT 16		• RHT 4
	↓ LHT 27		↓ LHT 27		↓ 0
4	↑ LHT 14	RE: rev IR/IO	↑ LHT 16	RE: rec IR 4.0 mm; rec SO; SR faden + rec SR 2.0 mm	↑ LHT 6
	• 0		• 0		• 0
	↓ RHT 18		↓ RHT 22		↓ RHT 4
5	↑ LHT 16	RE: rev + rec IR 3 mm	↑ LHT 12	RE: rec IR 3.0 mm; rec SO; SR faden	↑ 0
	• LHT 8		• 0		• 0
	↓ 0		↓ RHT 12		↓ LHT 2
6	↑ RHT 20	LE: rev + rec IR 4 mm	↑ RHT 16	RE: rec IR 3.5 mm; rec SO; SR faden + rec SR 2.0 mm	↑RHT 10
	• RHT 12		• RHT 2		• RHT 2
	↓ 0		↓ RHT 16		↓ LHT 4
7	↑ RHT 10	LE: rev + flap repair	↑ RHT 10	RE: rec IR 3.0 mm; rec SO; SR faden	↑ 0
	• LHT 14		• LHT 2		• 0
	↓ LHT 20		↓ LHT 12		↓ 0
8	↑ LHT 5	LE: rev + flap repair	↑ LHT 10	RE: SR faden	↑ 0
	• RHT 5		• LHT 3		• 0
	↓ RHT 18		↓ 0		↓ RHT 3

*RHT*, right hypertropia; *LHT*, left hypertropia; *SR*, superior rectus muscle; *IR*, inferior rectus; *SO*, superior oblique muscle; *IO*, inferior oblique; *rec*, recession; *rev*, revision; *faden*, posterior fixation suture. ↑, upgaze; •, primary position; ↓, downgaze; ∆, prismatic diopters

Postoperatively, the subjective diplopia report revealed that 4 patients (50%) were diplopia free, 3 patient (37.5%) presented diplopia in extreme upgaze, and 1 patient (12.5%) in mid-upgaze, and adduction. None of the patients reported diplopia in the primary position neither downgaze.

The field of BSV (binocular single vision) loss has remarkably improved in all of the patients after strabismus procedures from mean preoperative BSV loss of 80.63 ± 17.4 to 8.75 ± 8.3. The values of preoperative and postoperative field of BSV loss are summarized in Table 3.

**Table 3:** Preoperative and postoperative assessment of the field of BSV^a^ loss (%)

Patient	Preoperative BSV loss (%)	Postoperative BSV loss (%)
1	90	10
2	100	10
3	100	25
4	90	5
5	60	0
6	55	15
7	80	0
8	70	5
Mean	80.63 ± 17.4	8.75 ± 8.3

^a^Binocular single vision

**Fig. 1:**
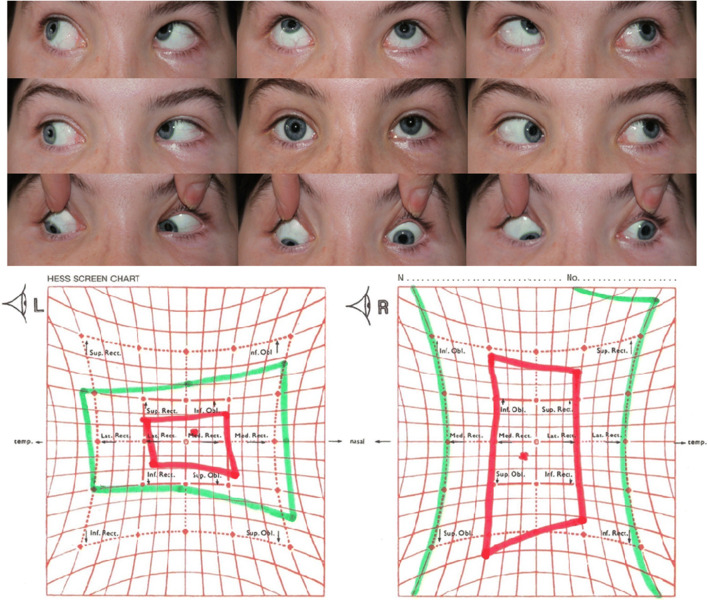
Ocular alignment and Hess chart before the I step surgery on the extraocular muscles in an exemplary patient (case 1). Trauma on the left orbit

**Fig. 2:**
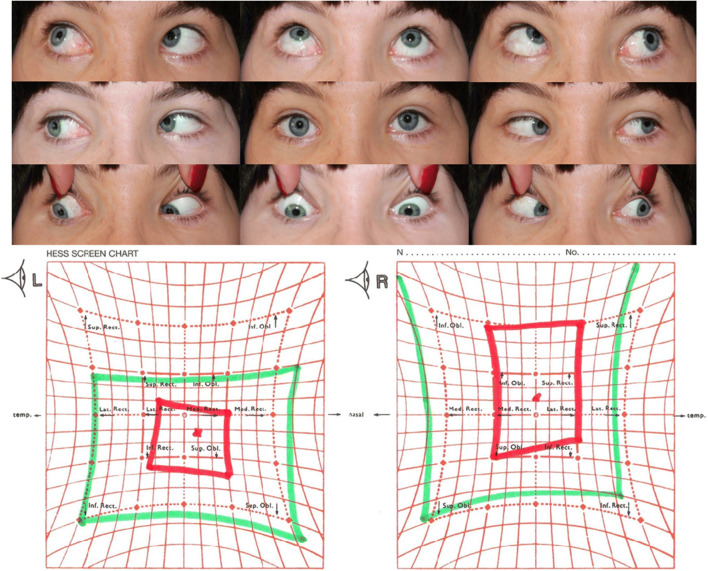
Ocular alignment and Hess chart before the II step surgery on the extraocular muscles in an exemplary patient. Trauma on the left orbit

**Fig. 3:**
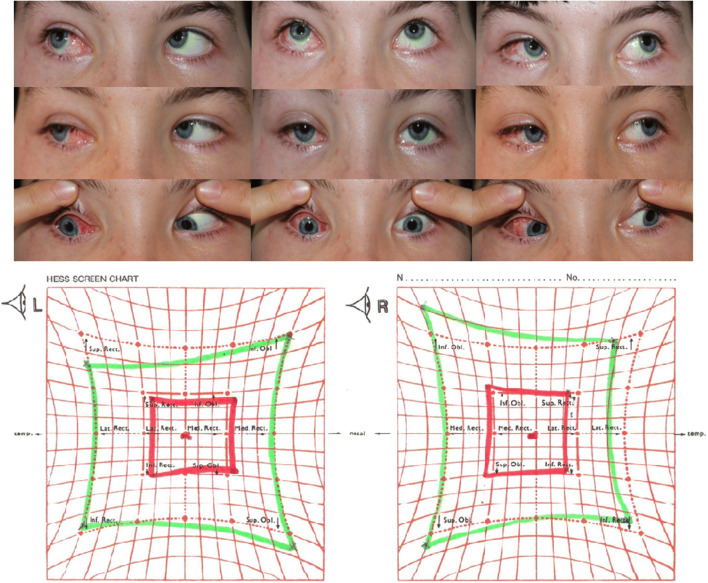
Final postoperative ocular alignment and Hess chart in an exemplary patient. Trauma on the left orbit

Ocular alignment and Hess chart before and after the surgeries on the extraocular muscles in an exemplary patient are presented in Figs. 1, 2, and 3.

## Discussion

Diplopia in upgaze accompanied by depression deficit is a complicated and challenging clinical problem. This is mainly due to a two-causal etiology of ocular motility impairment in patients that underwent reconstruction surgery for orbital fracture. The first cause is mechanical restriction related to adhesion formation, alloplastic implant malposition or not thorough revision of the fracture site by cranio-maxillo-facial surgeon. It might be accompanied by changes in muscle itself leading to scarring and fibrosis. If all the above affect the inferior rectus/inferior oblique complex, they result in limited elevation of the eye and diplopia in upgaze. The second cause is muscle paresis resulting from direct trauma to its belly (which is usually transient) that might have occurred during traumatic incident itself or later during reconstruction surgery. Especially when the maxillo-facial surgeon is not gentle enough while placing the alloplastic implant in the back of the orbit and the muscle is trapped in its posterior part which may result in limited elevation and diplopia in downgaze. A special issue is muscle being torn in a part from the side of the orbital wall. The so-called flap tear [10, 11] adheres to the surrounding tissues causing limited elevation along with depression in the mechanism of reverse leash.

Treating simultaneous elevation and depression deficit in patients after reconstruction surgery for orbital fractures is difficult as there are limited surgical options. The most challenging problem is not to alter ocular alignment in primary position as it is likely for this group of patients to see a single image while looking straight ahead. Despite thorough review of the literature, we have found just one paper concerning this topic [12]. This makes our study quite unique. Kushner proposed simultaneous recessions of the inferior and superior rectus muscles in order to enhance the freedom of movement of the affected eye. He recommended adjustable sutures to warrant the maintenance of single vision in primary position.

What we propose in such patients is to enlarge the area of binocular single vision by means of limiting the motility of the fellow eye. A perfect tool for such action is a faden operation (also known as posterior fixation suture) first described by Adelstein and Cüppers. It is performed by suturing the rectus muscle to sclera, 12–14 mm posterior to the rectus muscle insertion. Because of its progressive weakening effect when the eye is rotated towards the operated muscle, but does not change the resting position of the eye to any great extent, it can usefully be employed in order to reduce dissociated vertical divergence (DVD), to reduce convergence excess as well as to weaken the contralateral synergist in cases of muscle paresis [13]. The effect of any recession operation is enhanced when combined with the faden procedure [14]. This method was used in all of the patients from our study group by placing of a 14-mm posterior fixation suture on the contralateral superior rectus muscle, in order to restore vertical comitance in upgaze. In our series of cases, most of the patients presented mild to moderate limitation of elevation. Faden suture on contralateral superior rectus is known to have much poorer effect if the limitation on the affected side is severe [8]. That is why we have decided to begin with a revision of the traumatized inferior rectus/oblique complex. The synechiolysis combined with recession or repair of an encountered flap tear usually improved elevation. In consequence, the contralateral superior rectus faden operation effectively alleviated diplopia in upgaze.

In the next step, we had to deal with depression deficit which was or primary or resulted from a weakening procedure on the ipsilateral inferior rectus muscle. We have chosen to recess both contralateral inferior rectus and superior oblique muscles. Of course, one can consider another approach which is weakening of the inferior rectus muscle with faden suture. It seems that this might work better as the weakening effect would increase progressively in downgaze. However, it is problematic to place faden suture on the inferior rectus muscle due to the necessity of placing sutures close to vortex veins and close passage of inferior oblique which can easily get involved. It is worth emphasizing that in order to be effective, faden suture has to be placed just before the muscle pulley which is further than 14 mm from the insertion for the inferior rectus muscle.

Recession of the inferior rectus muscle is an established treatment for vertical strabismus [15] which allows to effectively weaken the muscle. In 7 cases from our study (87.5%), a small recession (3.0 to 5.5 mm) was added during the procedures performed as the second step. The amount of recession was adjusted in accordance with the angle of deviation in primary position (none, up to 3Δ; 2 mm, 3Δ to 5Δ; 3 mm, 6Δ to 14Δ; 4 mm for 15Δ and more). Applied for the contralateral inferior rectus muscle (in hypertropia) and superior rectus muscle (in hypotropia), it allows to adjust the primary position alignment. Most of the patients enrolled in our study presented some hyper- or hypotropia in primary position. The most challenging cases are those (4 patients from our study group) in which orthotropia is present in primary position and the surgical goal is not to alter this state. A major complication following inferior rectus recession reported by other authors is progressive overcorrection [16, 17]. The risk of postoperative overcorrection following inferior rectus recession should be considered, but in the study of Scotcher et al. [18] and Loba et al. [8], undercorrection occurred more frequently than overcorrection. Our results are consistent with those of the abovementioned studies since no overcorrections were noted among patients evaluated in our study, and small-angle undercorrections in primary position were noted in 2 cases (25%). However, we are not able to predict the possibility the occurrence of late-onset overcorrection.

In patients with limited function of inferior rectus on the side of traumatized orbit, one encounters large overaction of a contralateral superior oblique muscle. Its recession is reported to be an effective procedure in reducing downshoots in adduction in patients with A-pattern strabismus [19, 20].

The amount of recession is constant (10 mm) which means suturing the anterior edge of the superior oblique muscle 4 mm posteriorly to the nasal insertion of the superior rectus muscle. Our results show that it effectively reduced depression in adduction in all cases.

It is still questionable which time period between the orbital reconstruction surgery and first surgical correction for diplopia is the most appropriate to achieve the best possible outcomes. In our study, the mean time that elapsed from the reconstruction surgery to the first strabismus procedure was 10.3 ± 5.5 months (6 to 24 months). Unfortunately, we could not compare the results of our research with other authors since we were not able to find any published studies investigating this issue.

Moreover, several limitations to this retrospective study need to be acknowledged. Due to the fact that in a period of 7 years, only 8 cases were identified, the number of patients in our study group was relatively small. Furthermore, the postoperative follow-up in this study ranged from 6 to 24 months. Future investigations with longer follow-up and larger group of patients are needed to determine long-term outcomes of treatment.

To conclude, diplopia persisting after reconstructive surgery of a fractured orbital floor may be corrected surgically using various techniques. The results of our study suggest that at least two surgical procedures seem to be indicated to achieve satisfactory outcomes, such as the reduction of vertical deviation together with the restoration of binocular single vision resulting in resolution of subjectively reported residual diplopia. In such cases, contralateral inferior rectus recession combined with superior oblique recession and superior rectus posterior fixation appears to be effective procedures for use. However, despite many modifications and improvements of extraocular muscle surgeries, it is still challenging to resolve diplopia persisting after posttraumatic orbital floor reconstruction. Moreover, further studies are necessary to investigate the significance of differences in time period between the orbital reconstruction surgery and first surgical correction for diplopia and to determine which surgical procedures to achieve the most satisfying outcomes. These findings would be important clinical implications for future practice.
